# Reproducibility and sensitivity to change of various methods to measure joint space width in osteoarthritis of the hip: a double reading of three different radiographic views taken with a three-year interval

**DOI:** 10.1186/ar1831

**Published:** 2005-10-05

**Authors:** Emmanuel Maheu, Christian Cadet, Marc Marty, Maxime Dougados, Salah Ghabri, Isabelle Kerloch, Bernard Mazières, Tim D Spector, Eric Vignon, Michel G Lequesne

**Affiliations:** 1 Service de Rhumatologie, Hôpital Saint Antoine, Paris, France; 2 4 Place Martin Nadaud, 75020 Paris, France; 3 Medical Director, Clinica et Statistica, Issy les Moulineaux, France; 4 Université René Descartes, Paris V, Rheumatology Department, Hôpital Cochin, Paris, France; 5 Statistician, Clinica et Statistica, Issy les Moulineaux, France; 6 Project Manager, Expanscience Labs, Courbevoie, France; 7 Service de Rhumatologie, Hôpital Rangueil, Toulouse, France; 8 St Thomas's Hospital, London, UK; 9 Service de Rhumatologie, Hôpital, Lyon, France; 10 Service de Rhumatologie, Hôpital Léopold Bellan, Paris, France

## Abstract

Joint space width (JSW) and narrowing (JSN) measurements on radiographs are currently the best way to assess disease severity or progression in hip osteoarthritis, yet we lack data regarding the most accurate and sensitive measurement technique. This study was conducted to determine the optimal radiograph and number of readers for measuring JSW and JSN. Fifty pairs of radiographs taken three years apart were obtained from patients included in a structure modification trial in hip osteoarthritis. Three radiographs were taken with the patient standing: pelvis, target hip anteroposterior (AP) and oblique views. Two trained readers, blinded to each other's findings, time sequence and treatment, each read the six radiographs gathered for each patient twice (time interval ≥15 days), using a 0.1 mm graduated magnifying glass. Radiographs were randomly coded for each reading. The interobserver and intraobserver cross-sectional (M0 and M36) and longitudinal (M0–M36) reproducibilities were assessed using the intraclass coefficient (ICC) and Bland–Altman method for readers 1 and 2 and their mean. Sensitivity to change was estimated using the standardized response mean (SRM = change/standard deviation of change) for M0–M36 changes. For interobserver reliability on M0–M36 changes, the ICCs (95% confidence interval [CI]) were 0.79 (0.65–0.88) for pelvic view, 0.87 (0.78–0.93) for hip AP view and 0.86 (0.76–0.92) for oblique view. Intraobserver reliability ICCs were 0.81 (0.69–0.89) for observer 1 and 0.97 (0.95–0.98) for observer 2 for the pelvic view; 0.87 (0.78–0.92) and 0.97 (0.96–0.99) for the hip AP view; and 0.73 (0.57–0.84) and 0.93 (0.88–0.96) for the oblique view. SRMs were 0.61 (observer 1) and 0.82 (observer 2) for pelvic view; 0.64 and 0.75 for hip AP view; and 0.77 and 0.70 for oblique view. All three views yielded accurate JSW and JSN. According to the best reader, the pelvic view performed slightly better. Both readers exhibited high precision, with SRMs of 0.6 or greater for assessing JSN over three years. Selecting a single reader was the most accurate method, with 0.3 mm precision. Using this cutoff, 50% of patients were classified as 'progressors'.

## Introduction

Osteoarthritis (OA) is the most common rheumatic disease, and is becoming a major public health problem with the ageing of the population and the growing incidence of obesity in developed countries [1]. Treatment aims both to reduce symptom severity and to prevent or slow down disease progression and activity. Many symptom-modifying therapies have been proposed with various levels of evidence (for a recent review, see Zhang and coworkers [2]). However, we still lack a disease-modifying therapy because there is no treatment with proven efficacy in preventing, stopping, or retarding the disease process [2]. The structural process in OA affects cartilage, which is decreased in quality and thickness. Other structures may be involved in the damage observed in OA, including subchondral bone, articular capsule, synovium, meniscus and soft periarticular tissues. Hip OA is very common. It affects about 10% of the general population aged 65–74 years [3]. The prevalence of symptomatic hip OA increases dramatically with age.

Several trials have been conducted to identify structure-modifying drugs in hip OA, but as yet no such agent has exhibited convincing efficacy in this regard. The structural progression of OA is currently assessed on plain radiographic views by measuring the joint space width (JSW) and joint space narrowing (JSN) over a period of time [4]. This assessment is at present based on chondrometry, as described by Lequesne [5-7]. Other methods have been proposed, such as digitalized chondrometry (i.e. measurement of JSW or joint space surface with computer assistance [8]). Good reliability and sensitivity have been demonstrated for both methods [9,10]. At present, manual chondrometry – measurement of JSW at the narrowest point using a 1/10 mm graduated magnifying glass – performed by trained readers is the most commonly used technique. It has been shown to be sensitive to change and able to detect minor changes such as 0.5 mm over a one or two year period [11,12].

Recently published expert consensus recommendations [13,14] advocate the use of manual or digitalized measurement of joint space at the narrowest point on plain radiographic views of the pelvis in trials of structure-modifying treatment. However, there remains uncertainty concerning the optimal view for performing the measurement (anteroposterior [AP] pelvic view, feet in internal rotation of 15°, target hip AP view, or oblique view, which was proposed by Lequesne and Laredo [15] to be the 'false profile') and the number of readers that should perform the measurements in such trials. In 1987 Altman and coworkers [16] recommended three readers, but no evidence has yet been reported to support whether one, two, or even three readers should perform the measurements. It has been documented that radiography should be carried out in the standing rather than in the supine position [17,18]. The oblique view and plain pelvic view were compared in a pilot study conducted in 50 patients [19]. The combination of both views allowed identification of JSN in an additional one-third of patients, but the study did not attempt to identify the most sensitive view for performing chondrometry in a structure-modification trial.

The present study aimed to answer the following questions. Which radiographic view of the hip provides the most accurate measurement of JSW and JSN progression in hip OA? Should future trials of the structure-modifying effect of a treatment employ one or two trained readers for optimal assessment of disease progression and reliability of JSW measurement in hip OA?

## Materials and methods

### Patients

Hip radiographs were obtained from patients included in the ERADIAS study – an ongoing randomized, three-year, prospective, multicentre, double-blind, placebo-controlled trial of avocado/soybean unsaponifiables in hip OA. The study was approved by the ethics review board of the Pitié-Salpétrière Hospital. Included were outpatient with symptomatic hip OA (according to the American College of Rheumatology criteria [20]), who were 45–75 years old and who had a manually measured JSW on plain AP pelvic radiograph of 1–4 mm at baseline. All patients gave written informed consent to participate in the trial. Radiographs were verified by an independent assessor before study entry to ensure that patients were affected by OA; to ensure that the JSW was between 1 and 4 mm and assign the patient to one of the two strata (see below); and to exclude patients with isolated posteroinferior JSN, identified on the oblique view.

### Selection of radiographs

Radiographs from 50 patients were selected at random from radiographs of patients who had completed the three-year duration of the trial on 13 July 2004. Patients in the trial were stratified at entry into two strata: those with baseline JSW below 2.5 mm and those with baseline JSW 2.5 mm or greater, in order to ensure that the whole spectrum of disease was represented. For each patient, the protocol was to obtain three different radiographic views each year: plain radiograph of the pelvis, and target hip AP view and oblique view (Lequesne's false profile). Radiographs performed at baseline and at month 36 (M36 ± 3 months) were selected. The number of sets of radiographs required in each stratum was 25.

### Radiographic techniques

All radiographs were obtained at a standard size of 1/1 with the patient in a weight-bearing position. The X-ray beam was orientated AP, horizontal, and perpendicular to the table. The distance between X-ray source and film was 100 cm. Pelvis radiographs were performed with 15 ± 5° internal rotation of the feet and with the X-ray beam directed at the upper edge of the pubis symphysis. For hip AP views, 15 ± 5° internal rotation of the foot was also required but the the X-ray beam was directed at the joint space (with fluoroscopy). Oblique views were obtained using the technique described by Lequesne [13]. Patients were positioned with the foot axis (second metatarsus) parallel to the inferior edge of the radiography table and with the X-ray beam directed at the joint space (fluoroscopy). A sketch of the feet on the ground was drawn on heavy-weight paper during initial radiography and was used to position the patient at each subsequent examination.

Radiation exposure for each patient was 0.7 mSv (milliSieverts) for the pelvic view, 0.3 mSv for the hip AP view and 0.3 mSv for the oblique view. According to current private ambulatory practice in France, the cost of each of these views is 24.30 Euro (rated Z15 each, a Z costing 1.62 Euro).

### Blinding process for radiographs

Two lists of randomization (one per stratum) were used to code radiographs (using an alphanumeric code). Different alphanumeric codes were assigned to radiographs for each reading in order to avoid any identification of a set of radiographs that had already been read (reading one: list numbers 1–50; reading two: list numbers 51–100). Readers were blinded to the time sequence; letters A or B were randomly assigned to code the time sequence (M0 [baseline] or M36) on radiographs. Therefore, each radiograph was identified both by a letter and a number. All coded films (three views at M0 and three at M36, yielding a total of six films) for a single patient were gathered in an envelope.

### Reading procedures

Two trained readers (CC and EM) measured JSW using a 0.1 mm graduated magnifying glass. For each radiograph they were unaware of patient's identity, drug assignment, time sequence of the radiographs and each other's findings. Each set of six radiographs was read twice with a minimum time interval of 15 days between the two readings. Each radiograph was read on a horizontally positioned light box in order to identify the location and take an accurate measurement of the narrowest JSW area. All six views for each patient were read at the same time. About 10 sets of radiographs were read during each reading session (60 radiographs). A break was planned during each session so as not to exceed more than 2 consecutive hours of reading. Altogether, 300 radiographs were read twice, giving a total of 600 radiographs read. For the pelvic view, the target hip (i.e. the hip responsible for the patient's inclusion in the trial) to be read was indicated by a mark made by those in charge of randomization and labelling of radiographs. Readings were done between 24 August 2004 and 5 October 2004 by the two readers.

### Measurement of joint space width

The JSW of the hip joint was measured at the narrowest point for each view, in accordance with a previously described method [6]. Briefly, the site of measurement was marked by the reader using a special pencil that produces removable marks. The interbone distance was measured at this site with the help of a 0.1 mm graduated magnifying glass directly applied to the radiographic film and reported on a specifically designed case report form. The mark was then removed by the reader. For the oblique view, measurement had to be performed in the anterior and upper part of the circumference between the femoral head and the acetabulum, because no significant articular cartilage thickness could be measured at the posteroinferior segment of the view, especially after patients with posteroinferior hip OA had been excluded from the trial.

### Data management

Data were checked and queries sent to each observer when appropriate. For the Western and Ontario MacMaster University (WOMAC) score calculation, rules provided by the author were used [21]. Double key data entry was performed between 2 September and 5 October 2004.

### Statistical analysis

Descriptive data were recorded at baseline for the 50 patients selected: age, gender, BMI, mean disease duration, WOMAC score and Lequesne's index [22]. The data from radiographic readings were presented for each view and for each observer (reader 1, reader 2 and mean of the two readers) for M0 and M36, and their difference (M36–M0) using descriptive statistics (number, mean, standard deviation [SD], minimum, and maximum). The number of hips exhibiting a joint space change of 0.5 mm or more and those with a change of 0.3 mm or more between M0 and M36 were calculated for each view. The metrologic measurements taken for each view and each reader are shown in Table 1.

Accuracy of JSW measurement evaluated by intraobserver and interobserver reproducibility was assessed using the intraclass coefficient of correlation (ICC) [23] and using the Bland–Altman plotting method [24], which indicates the smallest detectable difference (SDD; i.e. the amount of detectable change above the random measurement error). Estimates of ICC were derived in the framework of a two-way fixed effect model. The 95% confidence interval (CI) was estimated using the method described by Fleiss and Shrout [25]. Mean difference, SD of the difference, 95% CI approximation of bias, limits, and 95% CI of upper and lower limits of agreements between measures were calculated. Using the SDD, the proportions of patients who could be considered to be 'progressors' were calculated.

Sensitivity to change of radiographic measures was estimated based on differences in JSW between M36 and M0 (from reading 1) using the standardized response mean (SRM; mean change/SD of change). The 95% CI of SRM estimates were calculated using the Jackknife technique [26] using the software S-PLUS professional (S-PLUS 6 for Windows; Insightful Corp., Seattle, WA, USA).

Paired tests and limits of agreements were used for comparisons between views and observers. When the null hypothesis (i.e. normal distribution) was rejected, the paired Wilcoxon test was used.

## Results

One hundred and forty-eight patients were included in the clinical trial between 7 February 2000 and 31 July 2001. The dropout rate in this sample was 45.9% (68/148), leaving 80 patients who completed the three years of follow-up. Radiographs of 29 patients were rejected for the following reasons: radiographs not received (five patients); one view missing or not available (11); radiographs sent for duplication and meanwhile not available (4); M0 or M36 radiograph not performed at the right time (i.e. more than 1 month delay; 2); M36 radiograph not obtained within the predefined time limit (i.e. 36 ± 3 months; 3); and poor radiograph quality (4). Among radiographs for the remaining 51 patients (26 in the low stratum and 25 in the high stratum), one patient was excluded by a random process to keep 25 radiographs in each stratum. Descriptive clinical data for the 50 patients whose radiographs were selected are shown in Table 2.

General results of radiographic measurements for each view and each observer (the mean of observers 1 and 2 is considered a third observer) are summarized in Table 3.

### Interobserver reproducibility

Data (mean of differences at baseline [± SD] and M0–M36 changes, ICC values) are provided for each view in Table 4. ICC values were 0.80 for the pelvic view, 0.88 for the target hip AP view and 0.72 for the target hip oblique view, indicating a good interobserver reproducibility. There was a systematic bias between the two readers; specifically, JSW measurements for reader 2 were slightly but systematically higher than those of the reader 1.

### Intraobserver reproducibility

#### Cross-sectional intraobserver reproducibility of radiographic measurements at baseline

The mean differences between repeated measurements of baseline radiographs are given in Table 5 for each view. ICC values were very high for both readers on all three views.

#### Longitudinal intraobserver reproducibility of measurements of joint space changes between baseline and M36

**Figure 1:**
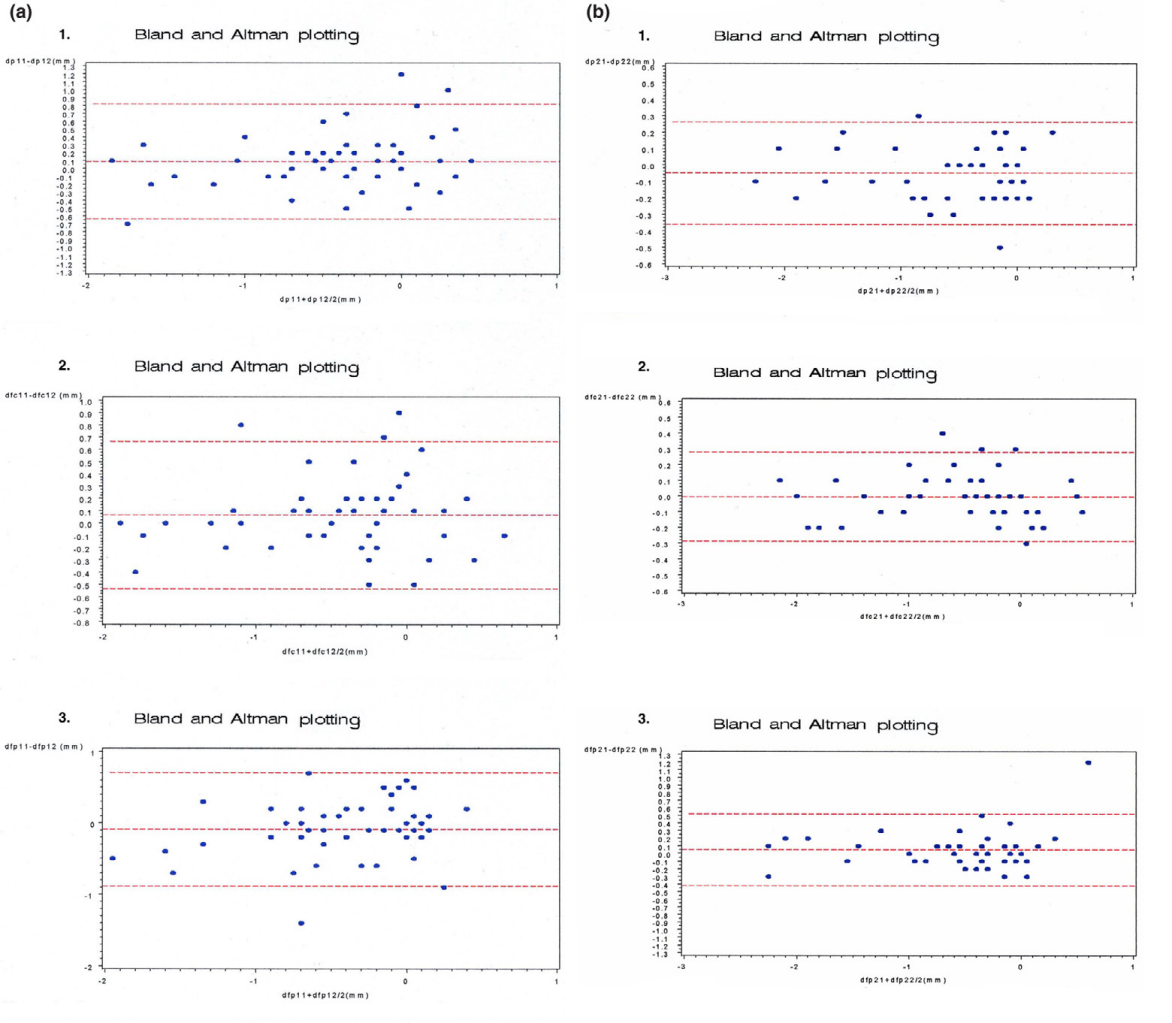
Intraobserver precision. Shown is the intraobserver precision, summarized using the Bland and Altman plotting method, for the assessment of changes in joint space width (JSW) between baseline (M0) and 36 months (M36) for the two readers and the three different radiographic views. A total of 50 sets of three radiographs taken at M0 and M36 were read twice by 2 readers with a 15 day interval. **(a) **Intraobserver reproducibility for reader 1 (M0–M36): 1, pelvic view; 2, hip anteroposterior (AP) view; 3, oblique view. **(b) **Intraobserver reproducibility for reader 2 (M0–M36): 1, pelvic view; 2, hip AP view; 3, oblique view.

The mean differences in repeated measurements of changes in JSW between baseline (M0) and M36 are given in Table 6 for each reader and each view. The Bland–Altman plotting method results for intraobserver reproducibility of measurements of changes between baseline and M36 are summarized in Fig. 1 for both readers and the three different views. ICC values were also very high for each observer for all three views, as shown in Table 6.

Both readers exhibited very good precision, as assessed using the ICC. Reader 2 was more accurate for all measures, as assessed both by ICC and Bland–Altman graphics (Fig. 1b). Adding a second reader or calculating the mean of the two readers did not confer any additional precision.

### Sensitivity to change over time

The SRM values were high, ranging from 0.61 (pelvic view, reader 1) to 0.82 (pelvic view, reader 2; Table 6). The estimate of the precision of the SRM calculated was performed using the Jackknife technique; 95% CI Jackknife SRMs are given in Table 6. According to values calculated in this study, radiographic measurement of JSW on the three views was sensitive. Reader 2 was more sensitive to change than was reader 1. All radiographic views appeared to provide similar levels of responsiveness. However, the pelvic view seemed to be the most sensitive in measuring changes in JSW – a basic property in trials of structure-modifying treatment.

JSW measurement is a continuous variable, and therefore it does not permit one to classify patients as disease progressors or nonprogressors. To translate this continuous variable into a dichotomous progression variable, we calculated the SDD, which can be derived using the Bland and Altman graphical approach. Its value is obtained by 2 SDs of the mean of differences between the two measurements. As may be calculated from data shown in Tables 5 and 6, the SDD for reader 2 was 0.32 mm for measurements of JSW and M0–M36 JSW changes on pelvic view and 0.30 mm and 0.28 mm, respectively, for measurements of JSW and M0–M36 JSW changes on the hip AP view.

The proportions of patients who could be classified as 'progressors' using the 0.3 mm cutoff or using the 0.5 mm cutoff previously described [11] are given in Table 7. Based on the reading precision offered by reader 2, the cutoff value of 0.3 mm was selected. Reader 1 identified 52%, 52% and 56% of progressors on pelvic, hip AP and hip oblique views, respectively. Reader 2 identified 48%, 54% and 52%, respectively. Using the 0.5 mm cutoff value, the respective proportions of progressors were 34%, 34% and 46% for reader 1, and 40%, 40% and 38% for reader 2.

Combining the results of measurements taken from the pelvic view and those taken from the oblique view led to a higher rate of identified progressors. Using the 0.3 mm cutoff, reader 1 identified 64% of progressors versus 52% on the pelvic view; using the 0.5 mm cutoff, 52% of progressors versus 34% were identified. The corresponding figures for reader 2 were 48% versus 48% and 46% versus 40%.

### Comparisons between views

The mean difference between the JSW measurements on pelvic and hip AP views was 0.01 ± 0.18 mm for reader 2 (at first reading), which was not statistically significant (*P *= 0.91 by Wilcoxon test). The mean difference for the same reader between the JSW measurements on pelvic and oblique views was 0.01 ± 0.64 mm, which was also not statistically significant (*P *= 0.89 by Student's t test). The study of correlations between measurements of M0–M36 JSW changes by reader 2 on pelvic and hip AP views exhibited very high correlation (Pearson correlation coefficient = 0.94; *P *< 0.0001).

## Discussion

Several radiographic views allow assessment of JSW and joint space changes in hip OA. To our knowledge, this is the first study to compare directly the metrologic measurement properties of JSW assessed using different radiographic views in hip OA obtained in the same sample of patients. Because the evaluation of a structure-modifying effect of a treatment is currently based on JSW measurement on radiographs, it is critical to optimize the technique used in order to maximize the precision of the measure. It must be noted that, in the present study, radiographs of poor quality or not performed within the predefined time limits from seven patients (9%) were excluded, which is not the procedure usually employed in clinical trial; instead, all radiographs are kept in such trial for use in an intent-to-treat analysis.

Our findings did not reveal significant differences between the ability of the different views to measure JSW reliably. With regard to intraobserver precision (either transversal at M0 or longitudinal between M0 and M36), and only considering the results for the better of the two readers, any of the three views could be used in a structural evaluation in hip OA because they yielded almost the same precision in assessment of JSW and joint space change. The limits of agreement at baseline ranged from -0.3 mm to +0.3 mm both for pelvic view and hip AP view, and for M0–M36 JSN measurement they ranged from -0.37 mm to +0.27 mm for the pelvic view and from -0.28 mm to +0.28 mm for the hip AP view. Cross-sectional and transversal intraobserver reproducibilities were consistent; the same values for dispersion (SD) were registered from the data for reader 2 from his readings of each of the three views at M0 and M0–M36 measurements.

In the present study interobserver reproducibility was less accurate than intraobserver (as shown in Tables 4, 5, 6). However, in the case of centralized reading performed by a single selected reader, it is clear that intraobserver precision is far more important than interobserver precision when examining the metrologic properties of an assessment tool aimed at measuring changes over time or with a given treatment.

The measurement of M0–M36 change in JSW provides an opportunity to assess the real measurement error. Indeed, it includes the error in measurement on a single radiograph (M0) along with the ability to detect change over time, and also includes the variability in measurement related to differences in patient repositioning at the second radiograph. When the aim is to select a tool to evaluate changes over time and/or to compare changes between groups, one must consider longitudinal intraobserver reliability and sensitivity to change, as given by the SRM. In the present study SRM values were good in all cases and for both readers. A SRM above 0.6 is considered good to excellent, whereas SRM values between 0.3 and 0.6 correspond to slight to moderate responsiveness. Unsurprisingly, measurements by the best reader provided the highest SRM values (ranging from 0.70 for the oblique view to 0.82 for the pelvic view). These values are consistent with SRMs calculated in previous studies comparing manual and digitalized assessment of joint space in hip OA [9,10].

Assessment of intraobserver precision provides an opportunity to calculate the SDD (i.e. the minimal amount of change that can be considered a change superior to the measurement error). The SDD allows determination of a cutoff value that segregates patients into those who had 'progressed' (i.e. lost cartilage thickness) and those who had not. This is of considerable importance in a trial in which the aim is to assess significant changes. The high precision in measurements by the second reader allowed us to select a 0.3 mm cutoff value, which is much lower than the 0.5 mm cutoff value usually recommended from previous studies [9,11]. Such a cutoff used in future clinical trials of structure-modifying treatment could result in increased statistical power and a reduction of the number of patients required. It would certainly permit a shorter duration of the trial (e.g. two years instead of three).

The present study shows that the precision of the measure is more dependent on the precision of the readers than on the radiographic view selected. Although the three views examined in the present study offered comparable precision in the assessment of JSW, either pelvic or hip AP view seems to be a good choice, offering a good reliability in measuring either JSW on a single view or joint space changes over time in pairs of radiographs taken 36 months apart. Although the best precision was obtained using the hip AP view, based on the Bland–Altman results and the SRM calculation, it may be more practical to choose the pelvic view (only slightly inferior to the hip AP view) because it also provides information on the contralateral hip.

The oblique view gives information that cannot be obtained when examining an AP view of the hip, even following exclusion of patients with isolated posteroinferior JSN, as was done in the present study. In a sample of hip OA patients with JSN in various locations, Conrozier and coworkers [19] showed that assessing the oblique in addition to the pelvic view resulted in identification of an additional 30% of patients with JSN. Our findings support the hypothesis that the combination of views could be superior to the use of a single view in identifying those patients whose joint space has changed. According to reader 2, 62% of patients could be classified as 'progressors' (i.e. patients exhibiting a decrease in JSW ≥ 0.3 mm) based on the combination of pelvic or oblique views, as compared with 48% of patients identified as progressors based on the pelvic view alone.

Using the pelvic or hip AP view, or combining one of them and the oblique view to assess structural modification in hip OA remains an option, depending on the trial aims and design. One could recommend that primary measurement of JS change be done using a single front view (either pelvic or hip AP) and that changes on both pelvic or hip face and oblique view be studied as secondary outcomes.

In France, in accordance with current ambulatory practice, the costs of each view were the same (costs are, of course, country dependent). The patients' radiation exposure was not very different between pelvic view, and hip AP or oblique views. Selection of the radiograph should not depend on such characteristics.

With regard to the number of readers that should be employed, our results conflict with previous recommendations that several readers be used [16]. A single reader was superior to the combination of two. Based on the results of this study, we recommend that the best reader be selected from among several trained readers before starting 'blinded' reading. This assumes that the reader has undergone preliminary training and that the reader will be selected to assess the primary outcome before the start of the trial. In our study we should like to identify two factors from among many possible explanations for the differences observed between the two readers, which could be taken into account in future trials: reader 2 was the most experienced of the two readers, having performed JSW assessment in several trials over the past 20 years; and furthermore, there were optical differences between the readers (reader 2 is a well corrected myopic and reader 1 a presbyopic). The latter factor leads reader 2 to remove his glasses when reading, with his myopia helping to magnify the image he reads. Optical impairments could be taken into account in the selection of readers; a myopic is preferable to a presbyopic.

## Conclusion

Our results show that the three radiographs usually performed in the radiographic examination of the hip offer good precision for assessment of JSW. However, pelvic or hip AP view allow more accurate measurement. The selection of one trained reader is preferable to using several readers in a trial. Furthermore, the better the precision of the reader, the fewer the patients required for the trial. A precision of 0.3 mm joint space change over time is attainable, using such procedures. When choosing this cutoff, 50% of the patients could be identified as 'progressors' in the sample selected in the present study, which would enhance statistical power greatly. Further investigations are required to compare digitized with manual chondrometry on these three views and joint space measurement on a single AP view versus the combination of AP and oblique views.

## Abbreviations

AP = anteroposterior; CI = confidence interval; ICC = intraclass coefficient of correlation; JSN = joint space narrowing; JSW = joint space width; OA = osteoarthritis; SD = standard deviation; SDD = smallest detectable difference; SRM = standardized response mean; WOMAC = Western and Ontario MacMaster University.

## Competing interests

The authors declare that they have no competing interests.

## Authors' contributions

EM devised the protocol of the trial and that of the present study with MM, performed the readings with CC, contributed to data analysis and wrote the manuscript. CC performed the readings and significantly contributed to the protocol development, data analysis and manuscript revision. MM devised the statistical section of the protocol, supervised the blinding process of radiographs, performed the randomization of radiographs, and verified the statistical analysis. MD significantly participated in devising the protocol of the study and in the analysis of data; he contributed to writing the manuscript and its revision. SG wrote with MM the statistical section of the protocol, checked the data and performed the statistical analysis. IK performed the follow-up of the trial and the radiographic study, and data management. BM, TS and EV significantly contributed to devising the protocol, analysis of data and revision of the manuscript. MGL was principal investigator of the trial; he significantly contributed to developing the design of the study, helped to define methods of measurement, and participated in manuscript development and revision.

## Figures and Tables

**Table 1:** Scheduled tested properties for each view and for each observer

Observer	Intraobserver reproducibility (M0)	Intraobserver reproducibility (M0–M36)	Interobserver reproducibility (M0)	Interobserver reproducibility (M0–M36)	Sensitivity to change (M0–M36)
Value from observer 1	x	x	x	x	x
Value from observer 2	x	x			x
Mean of values from observers 1 and 2	x	x			x
Data	Reading 1 and reading 2 of M0 for each observer	Difference between M0 and M36 for readings 1 and 2 for each observer	Reading 1 at M0 for two observers	Reading 1 for the difference between M0 and M36 for two observers	Difference between M0 and M36 in reading 1 for each observer

**Table 2:** Baseline characteristics of the 50 patients for whom radiographs were available

Baseline characteristic	Low stratum: JSW <2.5 mm (*n *= 25)	High stratum: JSW ≥ 2.5 mm (*n *= 25)	Total (*n *= 50)
Age (years; mean ± SD)	60.6 ± 8.2	59.9 ± 10.9	60.2 ± 9.6
Sex (*n *[%] female)	15/25 (60%)	14/25 (56%)	29/50 (58%)
BMI (kg/m^2^; mean ± SD)	27.5 ± 5.5	25.8 ± 3.0	26.6 ± 4.5
Disease duration (years; mean ± SD)	5.2 ± 4.5	3.6 ± 3.2	4.4 ± 4.0
Lequesne index (0–24; mean ± SD)	7.2 ± 2.0	6.9 ± 1.9	7.1 ± 2
WOMAC (total score: 0–100; mean ± SD)	35 ± 23.3	27.10 ± 15.75	31.12 ± 20.15
Global pain on VAS (mm; mean ± SD)	39.4 ± 25.8	35. 7 ± 15.1	37.6 ± 21.0

BMI, body mass index; JSW, joint space width; SD, standard deviation; VAS, visual analogue scale; WOMAC, Western and Ontario MacMaster University.

**Table 3:** General results of radiographic measurements

View	Observer	Reading	*n*	M0				M36				ΔM36–M0			
				
				Mean (mm)	SD	Min	Max	Mean (mm)	SD	Min	Max	Mean (mm)	SD	Min	Max
Pelvic	1	1	50	2.33	0.81	0.70	4.10	1.93	1.06	0.0	4.40	-0.40	0.65	-2.10	+0.80
		2	50	2.34	0.81	0.70	4.20	1.85	0.97	0.0	4.10	-0.49	0.54	-1.90	+0.40
	2	1	50	2.67	1.07	0.90	5.50	2.13	1.35	0.0	5.50	-0.54	0.66	-2.30	+0.40
		2	50	2.66	1.02	0.70	5.10	2.16	1.31	0.0	5.20	-0.49	0.67	-2.20	+0.20
	Mean (1 + 2)	1	50	2.50	0.91	0.70	4.65	2.03	1.17	0.0	4.95	-0.47	0.62	-2.05	+0.45
		2	50	2.50	0.89	0.70	4.50	2.01	1.11	0.0	4.55	-0.49	0.57	-2.05	+0.30

Hip AP	1	1	50	2.41	0.86	0.70	4.30	2.02	1.13	0.0	4.40	-0.39	0.61	-2.00	+0.60
		2	50	2.40	0.86	0.80	4.50	1.94	1.07	0.0	4.30	-0.46	0.59	-1.90	+0.70
	2	1	50	2.69	1.05	0.60	5.50	2.19	1.34	0.0	5.50	-0.49	0.66	-2.10	+0.50
		2	50	2.68	1.01	0.70	5.50	2.19	1.30	0.0	5.00	-0.49	0.66	-2.20	+0.60
	Mean (1 + 2)	1	50	2.55	0.94	0.65	4.90	2.11	1.22	0.0	4.95	-0.44	0.62	-2.05	+0.55
		2	50	2.54	0.92	0.75	5.00	2.06	1.17	0.0	4.55	-0.48	0.60	-1.95	+0.65

Oblique	1	1	50	2.37	0.79	0.70	3.70	1.92	1.05	0.0	3.80	-0.46	0.59	-2.20	+0.50
		2	50	2.29	0.78	0.40	3.60	1.92	1.02	0.0	4.10	-0.37	0.50	-1.70	+0.70
	2	1	50	2.69	1.00	0.50	5.00	2.21	1.28	0.0	5.00	-0.47	0.67	-2.40	+1.20
		2	50	2.70	1.01	0.60	5.00	2.18	1.27	0.0	5.00	-0.52	0.64	-2.30	+0.20
	Mean (1 + 2)	1	50	2.53	0.85	0.75	4.15	2.07	1.13	0.0	3.95	-0.46	0.61	-2.15	+0.65
		2	50	2.50	0.87	0.50	4.20	2.05	1.13	0.0	4.95	-0.45	0.53	-1.95	+0.35

Shown are the general results of radiographic measurements of joint space width (JSW) at baseline (M0) and 36 months (M36), and the joint space change for the three views and two observers (and their mean and SD). AP, anteroposterior; Δ, difference; SD, standard deviation.

**Table 4:** Interobserver reproducibility of joint space width measurements

View	Observer	Baseline JSW reading 1 (mm; mean ± SD)	Mean of difference (mm; mean ± SD)	ICC (95% CI)	Joint space change from M0 to M36 in reading 1 (mm)	ICC (95% CI)
Pelvic	1	2.33 ± 0.81	-0.34 ± 0.52	0.80 (0.52–0.91)	-0.40 ± 0.65	0.79 (0.65–0.88)
	2	2.67 ± 1.07			-0.54 ± 0.66	

Hip AP	1	2.41 ± 0.86	-0.27 ± 0.39	0.88 (0.66–0.95)	-0.39 ± 0.61	0.87 (0.78–0.93)
	2	2.69 ± 1.05			-0.49 ± 0.66	

Oblique	1	2.37 ± 0.79	-0.31 ± 0.61	0.72 (0.50–0.85)	-0.46 ± 0.59	0.86 (0.76–0.92)
	2	2.69 ± 1.00			-0.47 ± 0.67	

Shown is the interobserver reproducibility of joint space width (JSW) measurements between the two readers for the three radiographic views. AP, anteroposterior; CI, confidence interval; ICC, intraclass coefficient of correlation; SD, standard deviation.

**Table 5:** Cross-sectional intraobserver reproducibility of joint space width measurements at baseline

View	Observer	JSW (mm; mean ± SD)		Mean difference (mm; mean ± SD)	Limits of agreements	ICC values (95% CI)
					
		Reading 1	Reading 2			
Pelvic view	1	2.33 ± 0.81	2.34 ± 1.07	-0.01 ± -0.24	-0.49 to +0.47	0.96 (0.93–0.98), 0.99
	2	2.67 ± 0.81	2.66 ± 1.02	-0.02 ± 0.16	-0.30 to +0.34	(0.98–0.99) 0.98 (0.97–0.99)
	Mean (1 + 2)	2.50 ± 0.91	2.50 ± 0.89	0.004 ± 0.14	-0.28 to +0.28	

Hip AP	1	2.41 ± 0.86	2.40 ± 0.86	0.01 ± 0.25	-0.49 to +0.51	0.96 (0.93–0.98), 0.99
	2	2.69 ± 1.05	2.68 ± 1.01	0.01 ± 0.15	-0.29 to +0.31	(0.98–0.99) 0.98 (0.97–0.99)
	Mean (1 + 2)	2.55 ± 0.94	2.54 ± 0.92	0.01 ± 0.16	-0.30 to +0.32	

Oblique	1	2.37 ± 0.79	2.29 ± 0.78	0.08 ± 0.37	-0.89 to +0.71	0.88 (0.80–0.93), 0.98
	2	2.69 ± 0.78	2.70 ± 1.01	-0.02 ± 0.21	-0.41 to +0.51	(0.96–0.99) 0.96 (0.94–0.98)
	Mean (1 + 2)	2.53 ± 0.85	2.50 ± 0.87	0.03 ± 0.21	-0.29 to +0.35	

Shown is the cross-sectional intraobserver reproducibility of radiographic measurements of joint space width (JSW) performed on baseline radiographs. CI, confidence interval; ICC, intraclass coefficient of correlation; SD, standard deviation.

**Table 6:** Longitudinal (M0–M36) intraobserver reproducibility and sensitivity to change of joint space width measurements

View	Observer	JSW change (mm; mean ± SD)		Difference (mm; mean ± SD)	95% CI approximation of bias	Limits of agreements	ICC value (95% CI)	SRM calculated on reading 1 (95% CI)
							
		Reading 1	Reading 2					
Pelvic	1	-0.40 ± 0.65	-0.49 ± 0.54	0.09 ± 0.36	-0.01 to +0.19	-0.63 to +0.81	0.81 (0.69–0.89)	-0.61 (-0.65 to -0.57)
	2	-0.54 ± 0.66	-0.49 ± 0.67	-0.05 ± 0.16	-0.09 to -0.01	-0.37 to +0.27	0.97 (0.95–0.98)	-0.82 (-0.84 to -0.80)
	Mean (1 + 2)	-0.47 ± 0.62	-0.49 ± 0.57	0.02 ± 0.18	-0.2 to +0.06	-0.34 to +0.38	0.95 (0.92–0.97)	-0.76 (-0.77 to -0.73)

Hip AP	1	-0.39 ± 0.61	-0.46 ± 0.59	0.07 ± 0.30	-0.01 to +0.15	-0.53 to +0.67	0.87 (0.78–0.92)	-0.64 (-0.67 to -0.61)
	2	-0.49 ± 0.66	-0.49 ± 0.66	-0.00 ± 0.14	-0.04 to +0.04	-0.28 to +0.28	0.97 (0.96–0.99)	-0.75 (-0.77 to -0.73)
	Mean (1 + 2)	-0.44 ± 0.62	-0.48 ± 0.60	0.03 ± 0.16	-0.01 to +0.07	-0.29 to +0.35	0.96 (0.94–0.98)	-0.72 (-0.74 to -0.70)

Oblique	1	-0.46 ± 0.59	-0.37 ± 0.50	-0.09 ± 0.40	-0.21 to +0.03	-0.89 to +0.71	0.73 (0.57–0.84)	-0.77 (-0.80 to -0.74)
	2	-0.47 ± 0.67	-0.52 ± 0.64	0.05 ± 0.23	-0.10 to +0.11	-0.41 to +0.51	0.93 (0.88–0.96)	-0.70 (-0.74 to -0.67)
	Mean (1 + 2)	-0.46 ± 0.61	-0.45 ± 0.53	0.03 ± 0.16	-0.10 to +0.07	-0.29 to +0.35	0.91 (0.85–0.95)	-0.76 (-0.79 to -0.73)

Shown is the longitudinal intraobserver reproducibility and sensitivity to change (standardized response mean [SRM]) of radiographic measurements of joint space width (JSW; change from baseline [M0] to M36) on 3 different views read by two observers (and their mean). AP, anteroposterior; CI, confidence interval; ICC, intraclass coefficient of correlation; SD, standard deviation.

**Table 7:** Proportion of patients considered to be progressors

View	Observer	Patients with ≥ 0.3 mm change between M0 and M36	Patients with ≥ 0.5 mm change between M0 and M36
Pelvic	1	52%	34%
	2	48%	40%
	Both	40%	28%

Hip AP	1	52%	34%
	2	54%	40%
	Both	46%	32%

Oblique	1	56%	46%
	2	52%	38%
	Both	46%	34%

Shown are the proportions of patients considered to be progressors, using two different smallest detectable differences (SDDs) as cutoff values for defining progression (0.3 mm and 0.5 mm) for the two readers and three views. AP, anteroposterior.
